# When is patient-specific lung shunt fraction necessary in ^90^Y selective internal radiation therapy of liver cancer?

**DOI:** 10.1093/radadv/umag007

**Published:** 2026-02-05

**Authors:** Matthew Allan Thomas, Ryan C Lee, Tharun Alamuri, Dan Giardina, John Karageorgiou, Naganathan Mani, Daniel A Braga, Christopher D Malone

**Affiliations:** Mallinckrodt Institute of Radiology, Washington University School of Medicine, St. Louis, MO 63110, United States; Renaissance School of Medicine, Stony Brook University, Stony Brook, NY 11794, United States; Renaissance School of Medicine, Stony Brook University, Stony Brook, NY 11794, United States; Mallinckrodt Institute of Radiology, Washington University School of Medicine, St. Louis, MO 63110, United States; Mallinckrodt Institute of Radiology, Washington University School of Medicine, St. Louis, MO 63110, United States; Mallinckrodt Institute of Radiology, Washington University School of Medicine, St. Louis, MO 63110, United States; Mallinckrodt Institute of Radiology, Washington University School of Medicine, St. Louis, MO 63110, United States; Mallinckrodt Institute of Radiology, Washington University School of Medicine, St. Louis, MO 63110, United States

**Keywords:** lung shunt fraction (LSF), lung dose, ^90^Y, radioembolization, selective internal radiation therapy (SIRT), radiation segmentectomy, macroaggregated albumin (MAA) single photon emission computed tomography (SPECT)/CT, streamlined “no-MAA” ^90^Y

## Abstract

**Background:**

Lung shunt fraction (LSF) derived from macroaggregated albumin (MAA)-based nuclear medicine imaging is a standard component of yttrium-90 selective internal radiation therapy (^90^Y-SIRT) treatment planning. Elimination of MAA-based LSF determination has been suggested in selected cases.

**Purpose:**

To propose and evaluate a pretreatment identification method for patient-specific LSF that may influence treatment planning in ^90^Y-SIRT and necessitate LSF determination using MAA-based imaging.

**Methods:**

MAA SPECT/CT-based LSF (LSF_SPECT_) was analyzed retrospectively in glass ^90^Y-SIRT cases from September 2022 to June 2025 at a single center. A new metric (LSF_bound_) was defined as the minimum LSF value where the maximum achievable perfused volume (PV) dose is determined by a selected lung dose threshold (Lungs_max_) instead of a designated whole-liver dose threshold (Liver_max_). LSF_bound_ values computed using both clinical and simulated treatment planning parameters were quantitatively evaluated relative to LSF_SPECT_. A clinical workflow based on this new metric was evaluated.

**Results:**

A total of 354 cases were analyzed from 297 patients (92 females and 205 males). Median (interquartile range) age at MAA-SPECT/CT was 69 (63-74). LSF_bound_ depends only on liver mass, lung mass, Liver_max_, and Lungs_max_, whereas PV size plays no role. Using observed LSF_SPECT_ distributions, the median (max) probability for LSF_SPECT_ to exceed LSF_bound_ was ≤1% (≤4%) for hepatocellular carcinoma ≤ 8 cm and non-hepatocellular carcinoma cases without macrovascular invasion (87% of all cases). Receiver operating characteristic analysis showed that pretreatment use of LSF_bound_ could achieve 100% sensitivity and >60% specificities at Liver_max_ values up to 180 Gy.

**Conclusion:**

Patient-specific, MAA-based LSF determination may be obviated in most ^90^Y-SIRT cases as LSF and Lungs_max_ play no role in limiting the achievable PV dose. Pretreatment calculation of LSF_bound_ provides individualized, quantitative guidance for identifying when MAA-based, patient-specific LSF assessment is warranted.

**Summary** 

A pretreatment planning metric called LSF_bound_ shows that in most ^90^Y-selective internal radiation therapy cases, lung shunt fraction and lung dose do not limit the achievable perfused volume dose.

**Key Results** 

- Probabilistic distributions of lung shunt fraction (LSF) in ^90^Y-selective internal radiation therapy (^90^Y-SIRT) vary by cancer type and tumor size
- Liver/lung masses and liver/lung dose thresholds define LSF_bound_: the LSF value where the lung dose threshold starts to limit achievable perfused volume dose
- Tumors ≤8 cm without macrovascular invasion maintain a median (max) probability of <1% (4%) for clinical LSF to exceed LSF_bound_
- Pretreatment LSF_bound_ helps identify when patient-specific LSF is needed in ^90^Y-SIRT

## Introduction

The standard method for estimating lung dose (Lungs_Rx_) before yttrium-90 selective internal radiation therapy (^90^Y-SIRT) uses planar imaging for lung shunt fraction (LSF_planar_) and a nominal 1-kg lung mass.^1–3^ This approach results in biased, uncertain, and potentially misleading dosimetry values in many cases.^4–9^  ^99m^Tc-tagged macroaggregated albumin (MAA) SPECT/CT has been consistently shown^10–13^ to offer more accurate assessments of LSF (LSF_SPECT_), providing evidence that most ^90^Y-SIRT cases do not invoke high Lungs_Rx_^8^^,^^9^ or radiation pneumonitis.^14^ Modern ^90^Y-SIRT practices also involve treatment volumes and administered activities (AA) much smaller than those of historical whole liver or lobar approaches.^3^^,^^15^^,^^16^ Particularly for radiation segmentectomy of hepatocellular carcinoma (HCC),^3^^,^^17^^,^^18^ MAA-based LSF determination may not be necessary.

These combined effects led to the initial suggestion,^19^ and then prospective assessment,^20–24^ of a streamlined workflow where the MAA injection and related nuclear medicine imaging to estimate LSF are omitted completely from the planning procedure. The traditional angiographic “mapping” still takes place, but a nominal LSF value is assumed for treatment planning (e.g., 4%-5%). ^90^Y microsphere administration for therapy occurs in the same procedure. To date, the “no-MAA” workflow has been targeting early-stage, solitary HCC < 3 cm without macrovascular invasion (MVI)^25^ because even LSF_planar_, which is generally an overestimation compared to LSF_SPECT_, is low in such cases.^20–23^ Comparable tumor response and adverse event profile to traditional ^90^Y workflows,^21–23^ with reduced costs and enhanced patient access to care^24^ have been reported. For HCC ≥ 3 cm and non-HCC tumors, a “no-MAA” workflow may be theoretically possible but quantitative guidance is lacking.

In this work, we assessed the relationship between LSF_SPECT_ and a newly developed pretreatment planning metric termed LSF_bound_. LSF_bound_ is the minimum LSF value where a lung dose threshold (Lungs_max_), instead of the whole liver dose threshold (Liver_max_), restricts the achievable target dose. LSF_bound_ is predictive in nature, depending only on liver mass, lung mass, Lungs_max_, and Liver_max_. We retrospectively analyzed LSF_bound_ and probability distributions for LSF_SPECT_ across a large cohort of ^90^Y-SIRT cases. We demonstrated that, in a majority of cases, LSF_bound_ enables pretreatment assessment of whether a patient-specific, MAA-based LSF may influence treatment and therefore provide clinical benefit.

## Materials and methods

### Patient selection

**Figure 1:**
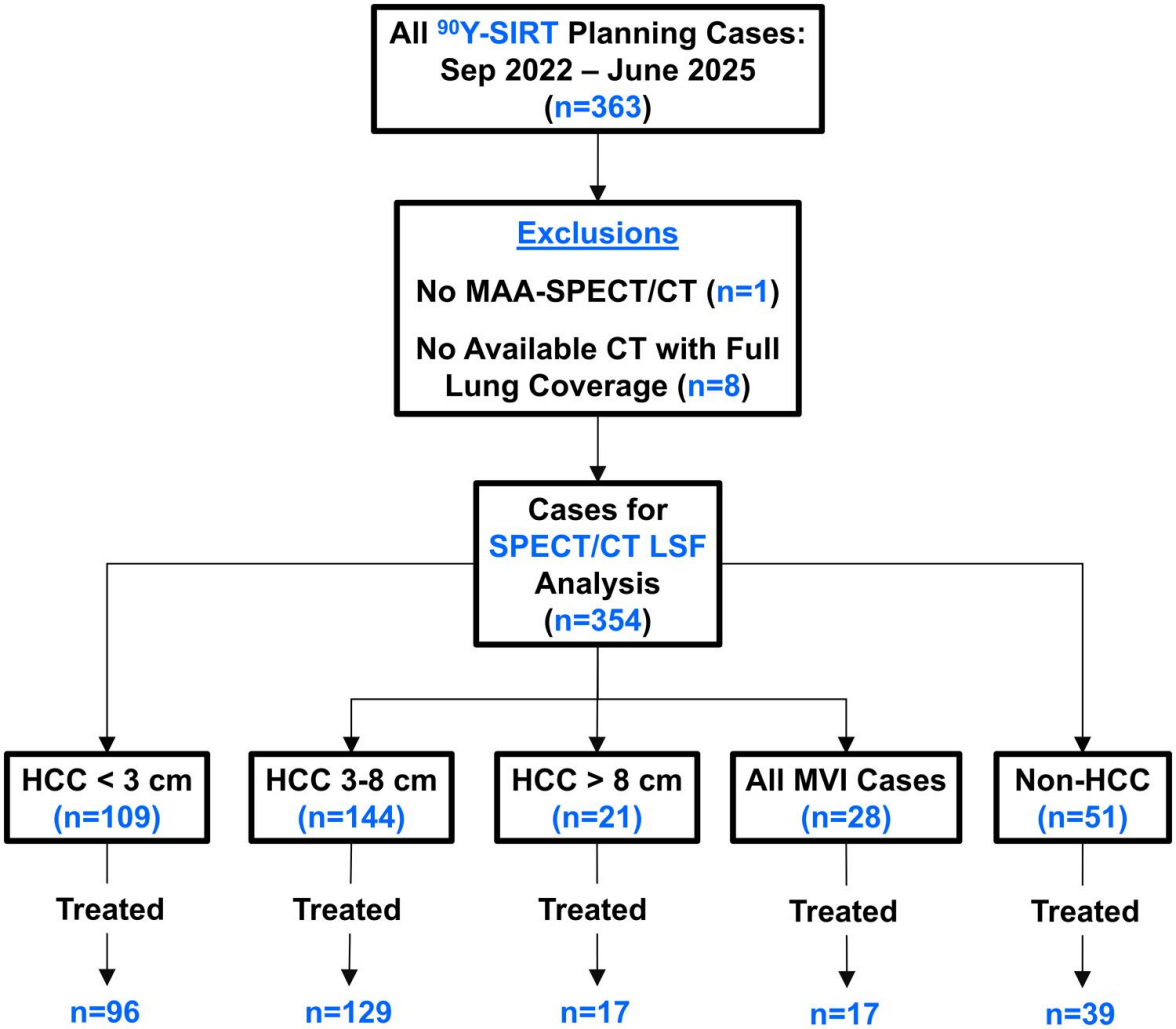
Case selection, exclusion, and categorization flowchart. From 363 eligible cases, 9 were excluded, 354 were used for LSF_SPECT_ analysis, and 298 proceeded to ^90^Y-SIRT. HCC = hepatocellular carcinoma; LSF = lung shunt fraction; MVI = macrovascular invasion.

This Health Insurance Portability and Accountability Act-compliant, retrospective study was approved by the local institutional review board with informed written consent waived. The same cohort previously studied for LSF analysis^9^ was used in this study (100% overlap). Consecutive liver SIRT cases using ^90^Y glass microspheres (Theraspheres) were collected from September 2022 to June 2025. Any case with a missing or nonusable MAA-SPECT/CT was excluded, and any case with no available CT with full lung coverage to estimate a patient-specific lung mass was excluded. Cases were categorized based on cancer type, maximum tumor diameter, and the presence of MVI as follows: (1) HCC < 3 cm, (2) HCC 3-8 cm, (3) HCC > 8 cm, (4) any case with MVI, and (5) non-HCC. Figure 1 outlines the case selection criteria and categorization.

### LSF analysis and clinical treatment planning

Details for LSF analysis and comparisons between LSF_planar_ and LSF_SPECT_ are outlined in a previous study.^9^ LSF_planar_ was used for all clinical treatment planning except in a small number of cases where LSF_SPECT_ was used instead (*n *= 11). A Lungs_max_ of 30 Gy (LSF_planar_) or 20 Gy (LSF_SPECT_)^5^ was used for clinical treatment plans. Only LSF_SPECT_ was used for analysis in this study and was computed using a 2-cm boundary correction at the liver/lungs interface to reestablish liver and lung counts.^7^^,^^26^ When present, lung truncation in the MAA-SPECT/CT field of view was corrected on a patient-specific basis as outlined previously.^26^ In this study, reported mean doses to the lungs (Lungs_Rx_), liver (Liver_Rx_), and perfused volume (PV_Rx_) were computed using single-compartment dosimetry, LSF_SPECT_, and patient-specific lung mass. Lung mass was estimated using patient-specific density and volume from CT, as in previous studies.^7^^,^^26^ See the Supplemental Information for more details on the necessity of using LSF_SPECT_ to report dosimetry in this study, as well as full treatment planning equations.

### PV_max_, TTR, and LSF_min_

The maximum achievable PV dose (PV_max_) is associated with 2 limiting thresholds: Liver_max_ and Lungs_max_. PV_max_ is the PV dose achieved when either Liver_Rx_ equals Liver_max_ or Lungs_Rx_ equals Lungs_max_. Two formulas for PV_max_ can be written in terms of either LSF/Lungs_max_ or Liver_max_:


(1) $${PV}_{\max}\;(Gy)=\;\frac{{\mathrm{Lungs}}_{\max}(Gy)\times {\mathrm{Mass}}_{\mathrm{lungs}}\;(kg)}{PV\;\mathrm{Mass}\;(kg)}\cdot \frac{\left(1-\mathrm{LSF}\right)}{\mathrm{LSF}}$$

(2) $${PV}_{\max}\;(Gy)=\;\frac{{\mathrm{Liver}}_{\max}(Gy)\times {\mathrm{Mass}}_{\mathrm{liver}}\;(kg)}{PV\;\mathrm{Mass}\;(kg)}$$

Lung and liver dose thresholds, lung and liver sizes, and LSF all combine to dictate the *PV_max_* in a specific case scenario. PV size (volume) determines the *specific PV_max_* value for a given case, but it is *not a factor* in determining what limits *PV_max_* because PV mass is common to both versions of *PV_max_* in Equations (1,2). The minimum *LSF* where *Lungs_max_* starts to determine PV_max_ instead of Liver_max_ can also be derived from Equations (1,2) as follows:


(3) $${\mathrm{LSF}}_{\mathrm{bound}}(\%)=\;\frac{1}{(\mathrm{TTR}\times {\mathrm{ratio}}_{\mathrm{live}r_{\mathrm{lungs}}})+1}\times 100$$

In Equation (3), *ratio_liver_lungs_* is the *mass_liver_* to *mass_lungs_* ratio, whereas *TTR* is the toxicity threshold ratio (*Liver_max_*/*Lungs_max_*). As an example, TTR = 6 represents the combination of 120 Gy Liver_max_ and 20 Gy Lungs_max_. In modern ^90^Y-SIRT treatment planning, different values may be appropriate and used clinically for Liver_max_ and Lungs_max_, leading to a range of possible TTRs. The Supplemental Information includes more details for Equations (1-3).

LSF_bound_ was computed with Equation 3 using clinical ratio_liver_lungs_, TTR computed using the LSF_SPECT_-adjusted Liver_Rx_ for Liver_max_, and 20 Gy Lungs_max_. Estimated worst-case LSF_bound_ values were also computed for respective cases using the maximum TTR value observed in each tumor category. The appropriate probability density function (PDF) for LSF_SPECT_ based on tumor category^9^ was used to estimate the probability (P_LSF>LSFbound_) that LSF_SPECT_ would exceed the corresponding versions of LSF_bound_ computed for each case.

### LSF_bound_ predictive performance

For pretreatment assessment using LSF_bound_, Liver_Rx_ (and TTR) will not be known precisely. Simulated TTRs and corresponding LSF_bound_ values were computed using Liver_max_ values from 0 to 250 Gy. Receiver operating characteristic (ROC) analysis was then performed using a range of probability thresholds (P_thresh_) from 0 to 50% for binary classification of LSF_SPECT_ > LSF_bound_. The predictions were compared to the simulated clinical data and ROC results were determined using the minimum P_thresh_ that kept sensitivity and negative predictive value (NPV) = 1 to prevent false negatives. False negatives—failing to predict LSF_SPECT_ exceeds LSF_bound_—is the key error to avoid in this clinical scenario despite the potential for a higher rate of false positives. Specificity and false positives were assessed as a function of simulated Liver_max_ value for each tumor category and all cases overall.

### Statistical tests

Most datasets were determined to be nonnormally distributed with Anderson-Darling and Shapiro-Wilk normality tests. As a result, Kruskal-Wallis tests were employed and median and range used for comparisons. A false discovery rate correction^27^ was applied, with all *P* values reported after multiplicity adjustment. All statistical analyses were performed with GraphPad Prism 10.5.1 (GraphPad Software, San Diego, California, USA) and statistical significance was considered true for *P *< .05. Further details for the probability analyses are provided in the Supplemental Information.

## Results

### Treatment planning: clinical cases

**Figure 2:**
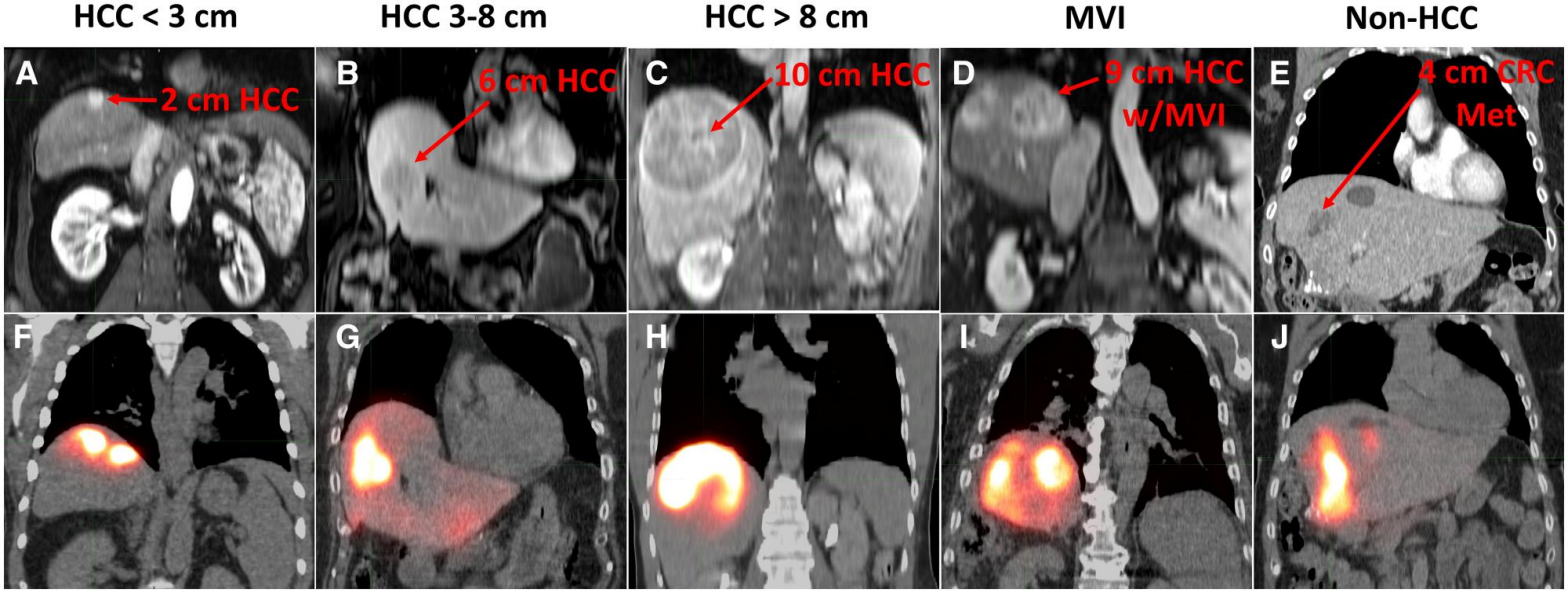
Morphological (A-E) and MAA-SPECT/CT (F-J) imaging for the example cases in each of the 5 tumor categories analyzed in this study. The target lesion and its size are identified in each morphological image. Full clinical parameter details for these 5 cases are included in Table 2 for reference. HCC = hepatocellular carcinoma; MAA = macroaggregated albumin; MVI = macrovascular invasion; CRC Met = colorectal cancer metastasis.

There were 354 cases with MAA-SPECT/CT data for LSF analysis, with 298 proceeding to ^90^Y-SIRT (Table 1). Figure S1 displays boxplots of much of the same data. Parameters like PV size, % liver treated, AA, Liver_Rx_, and Lungs_Rx_ all showed increasing trends as HCC tumor size also increased. PV_Rx_ decreased for larger tumors and non-HCC cases. Liver volume was higher for HCC >8 cm cases relative to all HCC ≤8 cm (*P *≤ .026) but not MVI and non-HCC tumors (*P *≥ .089). Mass_lungs_ was highly comparable across all groups, with no clear distinction for cancer type or tumor size (*P *≥ .99). Median values were ∼1 kg, the standard often used in treatment planning. Most cases had ratio_liver_lungs_ between 1.3 and 2.5, but some were as high as 3.0 in each of the tumor groups. Overall, females had a higher median ratio_liver_lungs_ of 1.88 compared to males at 1.69 (*P *= .028). Figure 2 shows morphological and MAA-SPECT/CT imaging from 1 example case in each tumor category. The clinical parameter details for these 5 cases are outlined in Table 2.

**Table 1:** Statistical summary of treated ^90^Y-SIRT cases (*n *= 298).

Patients	257 (180 males, 77 females)				
Age, years	Median: 69; IQR: 63-74; range: 29-96				
1 ^90^Y-SIRT	220				
2 ^90^Y-SIRT	33				
>2 ^90^Y-SIRT	4				
	HCC < 3 cm (*n *= 96)	HCC 3-8 cm (*n *= 129)	HCC >8 cm (*n *= 17)	MVI (*n* = 17)	Non-HCC (*n *= 39)
	Median (range)				
Liver volume (cm^3^)	1480 (690-3580)	1640 (755-3060)	2080 (1240-4460)	1490 (970-3120)	1810 (890-3360)
Lung mass (kg)	1.04 (0.60-1.60)	1.03 (0.54-1.54)	0.98 (0.50-1.42)	1.01 (0.71-1.46)	0.97 (0.65-1.53)
Tumor size (cm)	2.2 (1.2-2.9)	4.5 (3.0-8.0)	9.2 (8.2-14.7)	5.9 (1.2-10.6)	6.0 (0.9-11.8)
PV (cm^3^)	139 (19.6-1160)	227 (21.8-1420)	547 (203-1420)	405 (12.7-1080)	444 (37.7-2450)
% Liver treated	8.7 (1.5-75.6)	13.2 (1.6-77.8)	27.5 (12.8-72.9)	22.8 (0.9-44.4)	26.9 (2.8-92.7)
Ratio_liver_lungs_	1.6 (0.6-3.2)	1.8 (0.8-3.0)	2.0 (1.4-7.0)	1.7 (1.2-2.8)	2.0 (1.0-3.1)
AA (GBq)	1.63 (0.48-6.47)	2.37 (0.29-6.69)	4.00 (2.00-8.70)	2.32 (0.22-6.22)	2.56 (0.37-7.53)
PV_Rx_ (Gy)	569 (113-2640)	519 (110-2080)	342 (140-912)	411 (148-1690)	212 (69-2150)
Liver_Rx_ (Gy)	52.5 (17.1-118)	69.0 (10.8-191)	98.6 (41.4-272)	65.9 (7.4-164)	61.5 (19.3-151)
Lungs_Rx_ (Gy)	0.8 (0.1-8.2)	1.6 (0.1-25.1)	5.2 (0.3-22.5)	2.8 (0.1-18.3)	1.5 (0.1-20.7)
1 Vial	67	72	6	11	18
2 Vial	23	38	9	4	13
>2 Vial	6	19	2	2	8

Abbreviations: AA = administered activity; HCC = hepatocellular carcinoma; IQR = interquartile range; MVI = macrovascular invasion; PV = perfused volume; SIRT = selective internal radiation therapy.

**Table 2:** Clinical parameters for the example cases in Figure 2.

Parameter	HCC < 3 cm	HCC 3-8 cm	HCC >8 cm	MVI	Non-HCC
Figure 2 panels	(a, f)	(b, g)	(c, h)	(d, i)	(e, j)
Gender (age)	M (61)	F (70)	M (59)	M (74)	F (73)
Liver volume (cm^3^)	1120	2370	2100	1280	2100
Lung mass (kg)	1.08	1.20	0.89	1.01	0.82
Ratio_liver_lungs_	1.08	2.06	2.46	1.32	2.65
Tumor size (cm)	2.0	6.4	9.6	9.0	4.0
Liver segment(s)	7	4	7,8	8	4
PV (cm^3^)	95	330	780	540	360
PV_Rx_ (Gy)	505	410	305	150	310
Liver_Rx_ (Gy)	42	57	113	62	53
TTR	2.1	2.9	5.7	3.1	2.7
LSF_bound_ (%)^a^	30.5	14.6	6.7	19.6	12.6
LSF_SPECT_ (%)	2.7	1.2	7.5	9.3	2.3
P_LSF>LSFbound_	0	0	0.10	0.13	0.03

Abbreviations: HCC = hepatocellular carcinoma; MVI = macrovascular invasion; PV = perfused volume; TTR = toxicity threshold ratio.

a LSF_bound_ computed using retrospectively determined Liver_Rx_, rather than Liver_max_, and Lungs_max_ = 20 Gy.

### Clinically observed data: PV_max_, liver_max_, TTR, and LSF_bound_

Without consideration for LSF and Lungs_max_, PV_max_ is directly determined by % liver treated and the limit for Liver_Rx_ (Liver_max_). Smaller fractions of treated liver enable higher PV_max_. Figure S2 provides the clinical context for this relationship between PV_max_ and Liver_max_, both theoretically and for the 298 cases in this study. A total of 137 (46%), 99 (33%), 42 (14%), and 20 (7%) treated cases had Liver_Rx_ <60, 60-90, 90-120, and >120 Gy, respectively. These results confirm that most cases maintained a Liver_Rx_ <90 Gy. For cases with <15% treated liver, many had Liver_Rx_ <60 Gy (105/163, 64%). When combined with a theoretical Lungs_max_ of 20 Gy for LSF_SPECT_, the distributions of Liver_Rx_ values produced a range of clinical TTRs with characteristics tied to each tumor category as seen in Table 3. Median TTR increased as tumor size increased. Only HCC >8 cm, with some of the highest Liver_Rx_ values (>200 Gy at times), produced TTRs above 10.

**Table 3:** Clinical TTR, LSF_SPECT_, and LSF_bound_ statistics.

Parameter	HCC < 3 cm	HCC 3-8 cm	HCC >8 cm	MVI	Non-HCC
TTR median	2.6	3.4	4.9	3.3	3.1
TTR range	(0.9-5.9)	(0.5-9.6)	(2.1-13.6)	(0.4-8.2)	(1.0-7.5)
Max Liver_Rx_ (Gy)	118	191	272	164	151
LSF_SPECT_ median (%)	1.2	1.5	2.6	3.0	1.1
LSF_bound_ median (%)^a^	20.3	15.1	7.5	16.8	14.6
LSF_SPECT_ range (%)	(0.1-4.7)	(0.2-12.1)	(0.2-7.6)	(0.4-26.2)	(0.1-14.7)
LSF_bound_ range (%)^a^	(6.3-44.7)	(6.7-59.2)	(4.1-20.8)	(4.9-66.0)	(5.9-41.5)

Abbreviations: HCC = hepatocellular carcinoma; LSF = lung shunt fraction; MVI = macrovascular invasion; TTR = toxicity threshold ratio.

a LSF_bound_ computed using retrospectively determined Liver_Rx_, rather than Liver_max_, and Lungs_max_ = 20 Gy.

**Figure 3:**
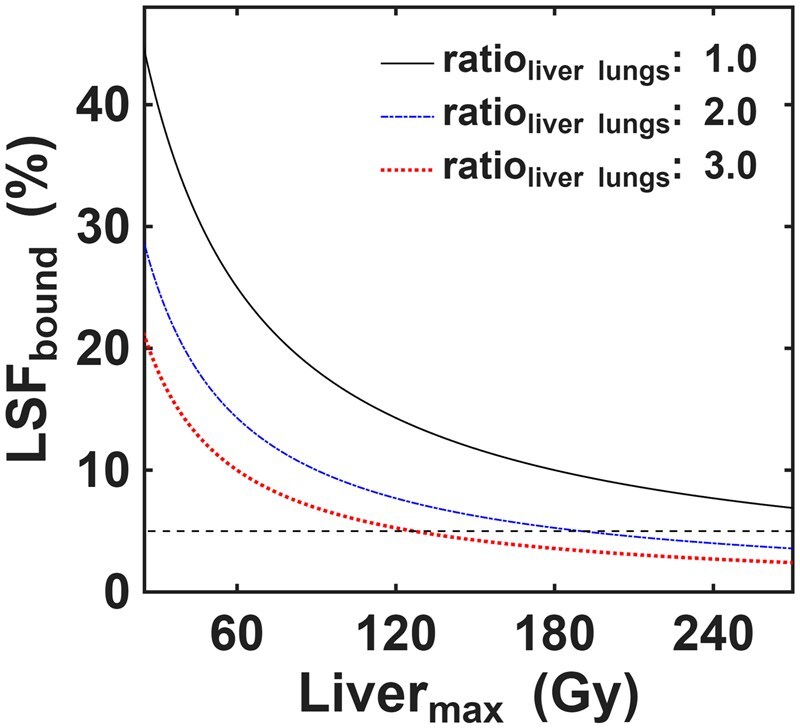
Simulated LSF_bound_ values as a function of Liver_max_ at 3 different ratio_liver_lungs_ ratios (1.0, 2.0, 3.0). A Lungs_max_ of 20 Gy was used. The dotted line represents an LSF_bound_ value of 5%. LSF = lung shunt fraction.

Figure 3 shows how LSF_bound_ changes as a function of Liver_max_ for 3 different ratio_liver_lungs_ values of 1.0, 2.0, and 3.0. These plots highlight that the Liver_max_ values required to yield an LSF_bound_ ≤5% are >270, >180, and >120 Gy for ratio_liver_lungs_ values of 1.0, 2.0, and 3.0, respectively. There were 26/354 (7%) cases in this study with LSF_SPECT_ >5%, 12/354 (3%) cases with ratio_liver_lungs_ >3.0, and 3 (1%) cases sharing both of these characteristics.

Table 3 includes summary data for clinical LSF_SPECT_ and LSF_bound_. The full distributions are shown in Figure S3. Median LSF_SPECT_ increased as tumor size increased. HCC >8 cm and cases with MVI maintained the highest LSF_SPECT_ values overall. Clinical LSF_bound_ values showed the opposite trend, with decreasing values as HCC tumor size increased. This was related to the higher ratio_liver_lungs_ that resulted from larger liver volumes (Table 1, Figure S1). HCC >8 cm cases had the lowest LSF_bound_ values among all groups (*P *≤ .002). There was no overlap between observed LSF_SPECT_ and LSF_bound_ values for HCC < 3-cm tumors. Other tumor categories showed some overlap, generally associated with outlier cases having the highest LSF_SPECT_ for each category.

Figure S4 shows distributions for P_LSF>LSFbound_, with detailed values included in Table 4. Specifically, probabilities were very low for all HCC ≤8 cm and non-HCC categories. For HCC >8 cm and cases with MVI, P_LSF>LSFbound_ was still clinically relevant with a moderate risk of occurrence. When LSF_bound_ was computed using the maximum TTR observed for each tumor category, probabilities increased slightly relative to those computed using exact clinical parameters. But even when using high TTRs derived from the extremes of each tumor category, median P_LSF>LSFbound_ was very low in HCC ≤ 8 cm and non-HCC tumors without MVI.

**Table 4:** Statistical summary: P_LSF>LSFbound_.

	HCC < 3 cm	HCC 3-8 cm	HCC >8 cm	MVI	Non-HCC
$P_{\text{LSF}>\text{LSFbound}}^{a}$					
Median	0	0	0.06	0.16	0.01
Range	(0-0)	(0-0.04)	(0-0.32)	(0-0.33)	(0-0.04)
$P_{\text{LSF}>\text{LSFbound}}^{b}$					
Median	0	0.06	0.37	0.25	0.04
Range	(0-0)	(0.01-0.18)	(0.22-0.76)	(0.20-0.39)	(0.02-0.10)

Abbreviations: HCC = hepatocellular carcinoma; MVI = macrovascular invasion; TTR = toxicity threshold ratio.

a LSF_bound_ computed using retrospectively determined Liver_Rx_, rather than Liver_max_, and Lungs_max_ = 20 Gy.

b LSF_bound_ computed using max TTR in each tumor category (Table 3).

### Pretreatment planning with LSF_bound_

**Figure 4:**
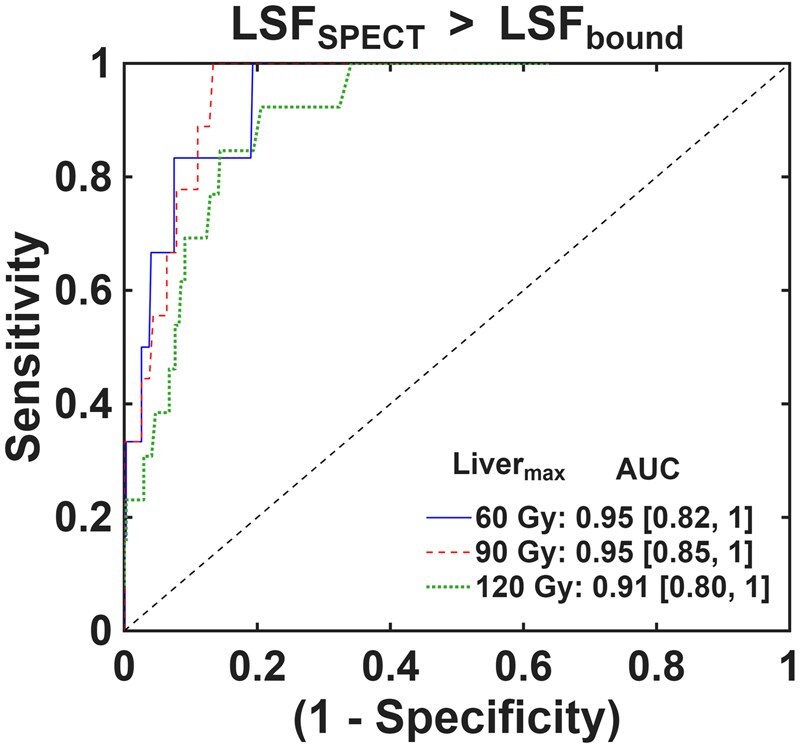
ROC plots for accurate prediction of LSF_SPECT_ > LSF_bound_ using a range for P_thresh_ from 0 to 50%. Three different Liver_max_ values of 60, 90, and 120 Gy were combined with Lungs_max_ of 20 Gy to establish LSF_bound_ and assess predictive performance. The equality line representing random prediction accuracy is included for reference. The area under the curve (AUC) with 95% confidence interval range is included for each Liver_max_ value. There were only 6, 9, and 13 cases with LSF_SPECT_ > LSF_bound_ using a simulated Liver_max_ of 60, 90, and 120 Gy, respectively. This means the simulated data was biased toward the few positives, making the optimal P_thresh_ value to avoid false negatives produce some false positives. LSF = lung shunt fraction; ROC = receiver operating characteristic.

**Figure 5:**
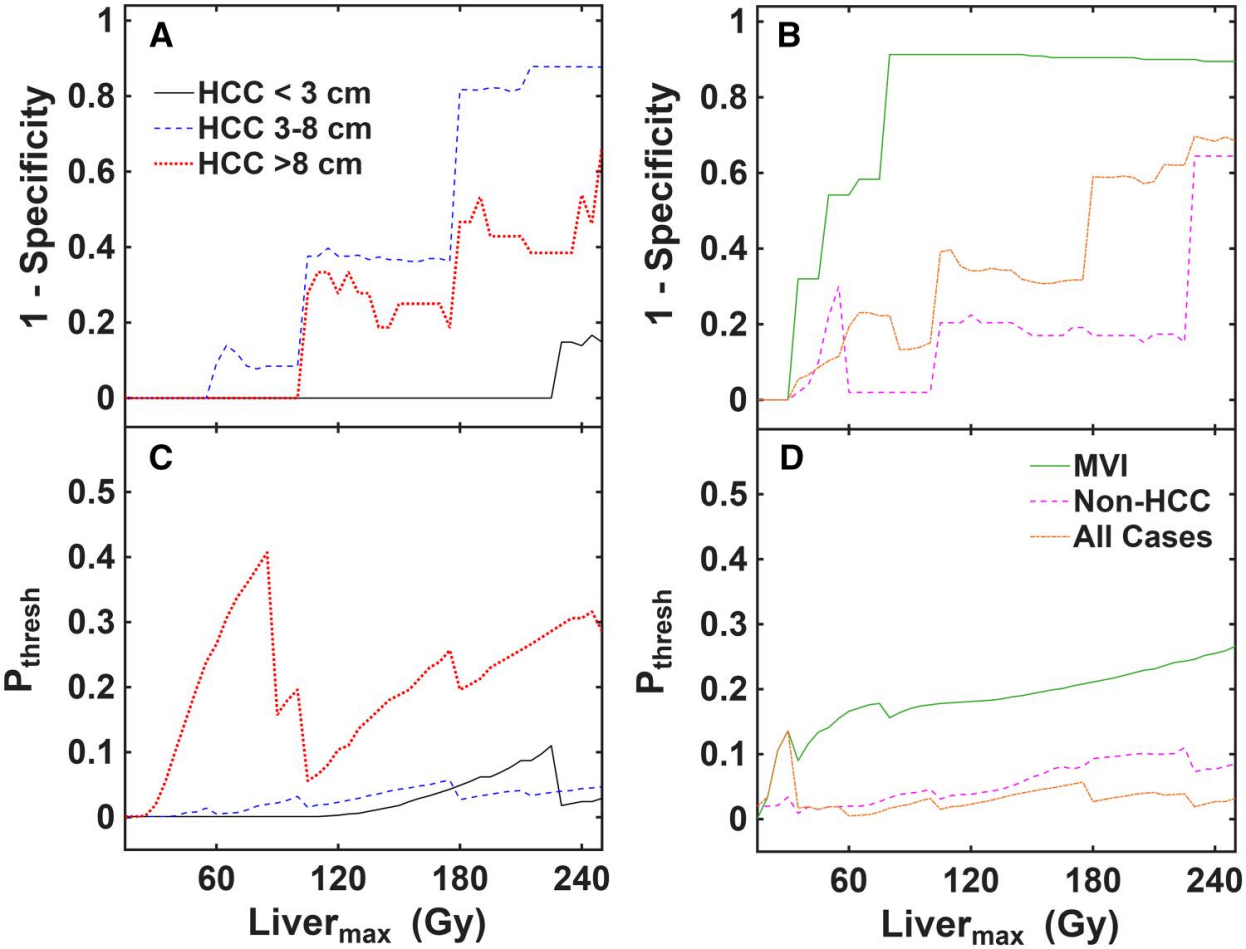
Plots of 1—specificity (false-positive rate; A, B) and optimal probability threshold (P_thresh_; C, D) as a function of Liver_max_ from the simulated LSF_bound_ and ROC analysis. HCC tumor categories are shown in (A, C) while MVI, non-HCC, and all cases combined are shown in (B, D). P_thresh_ values were associated with maintaining sensitivity and NPV both = 1 (no false negatives) while maximizing specificity. HCC = hepatocellular carcinoma; LSF = lung shunt fraction; MVI = macrovascular invasion; NPV = negative predictive value; ROC = receiver operating characteristic.

Traditional ROC curves using three Liver_max_ values of 60, 90, and 120 Gy applied across all cases globally are shown in Figure 4 to give a sense of the general predictive capabilities of LSF_bound_. Detailed ROC stats are included in Table S1 and discussed in the Supplemental Information. Generally, the LSF_bound_ approach yields excellent predictive results, with high area under the curve for each of the 3 Liver_max_ values assessed. Figure 5 shows detailed ROC results across all Liver_max_ values tested (0-250 Gy). As Liver_max_ increased, the false-positive rate for predicting LSF_SPECT_ > LSF_bound_ increased while maintaining no observed false negatives. This is because LSF_bound_ decreases as Liver_max_ increases (Equation 3). But the specific false-positive rate depended on the tumor category. HCC < 3 cm tumors only yielded false positives at very high (>200 Gy) Liver_max_, which is not clinically realistic for such cases. HCC 3 to 8 cm, HCC > 8 cm, and non-HCC tumors maintained 60% or higher specificity up to ∼180 Gy Liver_max_. MVI cases showed a high number of false positives at any Liver_max_ >70 Gy. The specific P_thresh_ values associated with these predictive results must also be considered, however. HCC >8 cm tumors and MVI cases generally required a higher P_thresh_ to yield optimal specificity. It would likely be difficult to employ such P_thresh_ values of 20% or higher in a clinical setting because of concern for reliable predictions. On the other hand, P_thresh_ values for all HCC ≤ 8 cm and non-HCC groups were generally much lower, often well below 10%. As an example, a P_thresh_ of well below 5% could be applied globally to all cases except the MVI group and achieve perfect sensitivity and ≥80% specificity up to a Liver_max_ of ∼90 Gy. For higher Liver_max_ values, more specific P_thresh_ values applied to each tumor category may be required for optimal performance.

**Figure 6:**
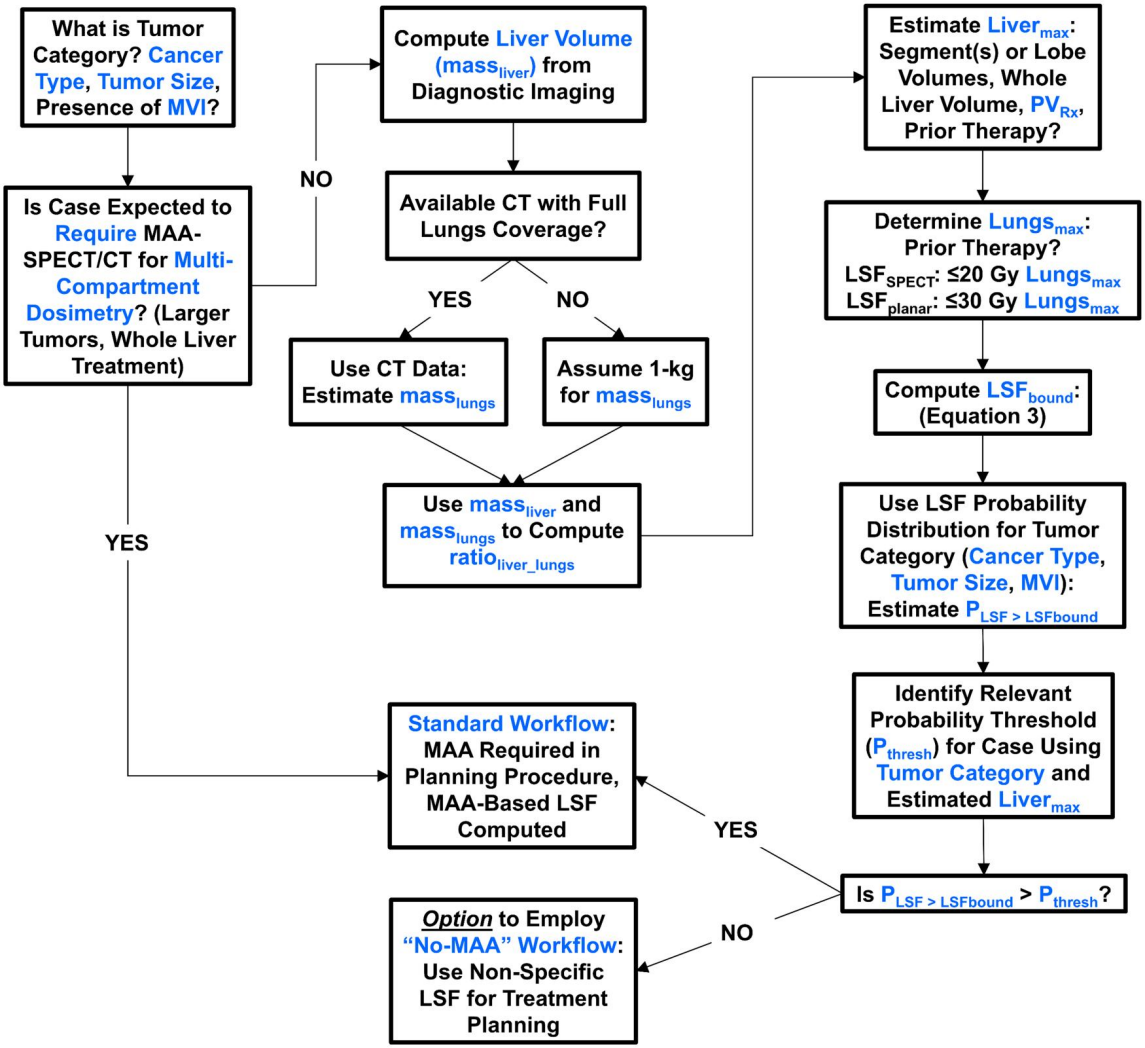
Proposed ^90^Y-SIRT treatment planning workflow based on the clinical data and analysis in this study. MVI = macrovascular invasion.

Figure 6 outlines a proposed treatment planning workflow for ^90^Y-SIRT informed by the results in this study. The exact PV size is not used in this methodology, so all steps can be completed before any ^90^Y-SIRT angiographic procedures. Clinical characteristics associated with cancer type, tumor size, and MVI status determine expectations for the applicability of single-compartment dosimetry. The proposed methodology applies only to such cases, where MAA-SPECT/CT will not be required to inform multicompartment dosimetry. PV size could be conservatively estimated using tumor location (ie, segment, lobe) to better understand the range of potential treatment volumes. These data would be combined with the whole liver volume and desired PV_Rx_ to produce estimations for Liver_max_. Liver_max_, Lungs_max_, and ratio_liver_lungs_ would then be used for pretreatment calculation of LSF_bound_. The case’s tumor category can inform probabilistic expectations for clinical LSF, enabling an estimate for P_LSF>LSFbound_. A global (all tumors) or tumor category-specific probability threshold for relevant risk (P_thresh_) is then employed to directly compare with P_LSF>LSFbound_. The necessity of including an MAA injection as part of the planning procedure to produce a patient-specific LSF is then assessed via comparison of P_thresh_ to P_LSF>LSFbound_. The example case scenarios from Figure 2 and Table 2 are discussed with respect to this proposed workflow in the Supplemental Information. Each institution may dictate its own preferred risk thresholds and specific usage of P_LSF>LSFbound_, P_thresh_, and elimination of MAA-based, patient-specific LSF.

## Discussion

This study proposes a new metric (LSF_bound_) to determine the necessity for patient-specific LSF determination using clinical parameters such as Liver_max_, mass_liver_, and mass_lungs_ that are available pretreatment. LSF and Lungs_max_ rarely restrict single-compartment ^90^Y-SIRT when the more accurate LSF_SPECT_ is used. The median (max) probability for LSF_SPECT_ to exceed LSF_bound_ was ≤1% (≤4%) for HCC ≤ 8 cm and non-HCC cases without MVI (87% of all cases). A clinical workflow was proposed based on these results.

Pretreatment use of LSF_bound_ requires an estimate for Liver_max_. Guidelines for PV_Rx_ could provide generalization for Liver_max_ in many cases. For example, a PV_Rx_ of 400 Gy for up to 15% liver treated aligns with a Liver_max_ of ≤60 Gy, whereas a PV_Rx_ of 600 Gy for up to 15% liver treated aligns with a Liver_max_ of ≤90 Gy. More than 50% of cases in this study (163/298) were associated with <15% liver treated, suggesting that 60 Gy or 90 Gy Liver_max_ values could be used prospectively in a majority of cases. Furthermore, only 20 (7%) cases had Liver_Rx_ >120 Gy. A Liver_max_ of 120 Gy could be used as a “worst case scenario” for pretreatment estimation of LSF_bound_ in all but the most unique of cases (very large liver or PV). Overall, the probability for clinical LSF to exceed the pretreatment-calculated LSF_bound_ is infrequent in practice. HCC >8 cm and tumors with MVI warrant extra attention because of their potential for high clinical LSF and P_LSF>LSFbound_ given higher Liver_max_ and lower calculated LSF_bound_.

The historical use of planar imaging for LSF has produced inaccurate and biased data that presents the *illusion* of LSF and Lungs_Rx_ being key aspects of patient selection and treatment planning. A more rational use of patient-specific LSF *when necessary* can enable a more informed patient selection and treatment planning paradigm. Nevertheless, estimating Lungs_Rx_ for each case is still important for documentation and informing future radiation-based treatments when indicated. If MAA injection is removed from planning, how can estimates for LSF and Lungs_Rx_ be determined? Recent work has shown that multiphase CT^28^ and MRI^29^ analyses of PVs and tumors strongly correlate with MAA-based LSF. A nominal, conservative LSF that is appropriately matched to the clinical characteristics of the case may also suffice, as employed in current no-MAA workflows.^21–24^ In particular, assuming an LSF of 5% for HCC < 3 cm despite a median LSF_SPECT_ of 1.2% and no case in this study reaching 5% for this tumor category (Table 3) has minimal impact for both PV_Rx_ and Liver_Rx_. More importantly, this strategy enables more aggressive PV_Rx_ at similar Lungs_Rx_ compared to that derived from the traditional use of LSF_planar_ because LSF_planar_ may be > 5% in *more than half* of cases with HCC < 3 cm.^9^

This study had limitations that should be addressed. Only LSF_SPECT_ was analyzed in this study, which is the more accurate and reliable approach for LSF calculation.^10–13^ But some institutions may still rely on LSF_planar_ for ^90^Y-SIRT patient selection and treatment planning. A comparative analysis of the LSF_bound_ methodology using LSF_planar_ is worthy of future study. Some of the tumor categories analyzed here maintained a small sample size, increasing the uncertainty of the probabilistic analyses and reducing the generalizability of the results for such tumor categories. This was a single-center study and the quantitative analyses applied were also generally limited by their probabilistic nature. The proposed clinical workflow supported by the use of LSF_bound_ may be difficult to adopt at all institutions. Further investigations for validation of the study’s methods at other institutions are therefore necessary to ensure consistent results for broad clinical use. No resin ^90^Y cases were included. Glass vs resin *plays no role* in MAA-based LSF assessments, but treatment volumes, target doses, etc. may differ between the 2 devices. The pretreatment approach using LSF_bound_ can be applied to resin ^90^Y-SIRT with appropriate choices for Liver_max_ and Lungs_max_. A criticism associated with removing the MAA injection and pretherapy nuclear medicine imaging in a large cohort of ^90^Y-SIRT cases is the inability to identify extrahepatic shunts. However, current ^90^Y-SIRT practice has transitioned to use modern angiographic and cone-beam CT techniques before ^90^Y microsphere administration as the gold standard for shunt identification.^19^^,^^30^

To conclude, a pretreatment methodology based on the newly proposed LSF_bound_ showed that patient-specific LSF provides minimal clinical benefit in a large number of ^90^Y-SIRT cases. Analyses using actual and simulated clinical parameters demonstrated that a workflow with pretreatment calculation of LSF_bound_ can identify patients who may forego MAA-based, patient-specific LSF determination. Future work will assess the prospective application of the LSF_bound_ approach to new clinical cases and other institutions whose data were not used to inform the quantitative models outlined in this study.

## Supplementary Material

umag007_Supplementary_Data

## Data Availability

Research data are stored in an institutional repository and will be shared upon request to the corresponding author.
