# Supplementary material for: When is patient-specific lung shunt fraction necessary in 90Y selective internal radiation therapy of liver cancer?
Source: Radiol Adv. 2026 Feb 5;3(2):umag007. doi: 10.1093/radadv/umag007 (PMC13005926; doi:10.1093/radadv/umag007)
Supplement: umag007_Supplementary_Data [file umag007_supplementary_data.zip › Supplemental File Only.pdf]

When is Patient-Specific Lung Shunt Fraction Necessary in ^{90}Y Selective Internal Radiation Therapy of Liver Cancer?

M. Allan Thomas[1], PhD, Ryan C. Lee[2], BS, Tharun Alamuri[2], BS, Dan Giardina[1], MD, John Karageorgiou[1], MD, Naganathan Mani, MD, Daniel A. Braga[1], MD, and Christopher D. Malone[1], MD

[1]Mallinckrodt Institute of Radiology, Washington University School of Medicine, St. Louis, MO 63130, USA

[2]Renaissance School of Medicine, Stony Brook University, Stony Brook, NY 11794, USA

Supplemental Information

Additional Materials and Methods Details

Some prior work [1], including our own [2], has included the presence of TIPS as a similar characteristic to MVI for segregating cases with risk for high LSF. In this study, we determined that cases only with TIPS and not also MVI (n=7) maintained similar LSF distributions as other tumors in their relevant category (HCC, non-HCC, tumor size). As a result, the only distinction used to categorize tumors in the MVI category was the presence of MVI. All cases with only TIPS were otherwise included in their relevant tumor category.

Using the Medical Internal Radiation Dosimetry (MIRD) formalism, the prescribed mean dose to the lungs ($Lungs_{Rx}$), perfused volume (PV; PV_{Rx}), and whole liver ($Liver_{Rx}$) in ^{90}Y-SIRT treatment planning can be written as follows:

$$Lungs_{Rx}\ (Gy) = \frac{49.67(\frac{Gy \cdot kg}{GBq}) \times AA(GBq) \times LSF}{Mass_{lungs}\ (kg)} \quad \text{(S1)}$$

$$PV_{Rx}\ (Gy) = \frac{49.67(\frac{Gy \cdot kg}{GBq}) \times AA(GBq) \times (1-LSF)}{PV\ Mass\ (kg)} \quad \text{(S2)}$$

$$Liver_{Rx}\ (Gy) = \frac{49.67\left(\frac{Gy\cdot kg}{GBq}\right)\times AA(GBq)\times(1-LSF)}{Mass_{liver}\ (kg)} \tag{S3}$$

In all three equations, AA is the prescribed administered ^{90}Y activity in GBq. The clinical treatment plans created for the cases in this study used the reference standard lung mass of 1-kg to compute $Lungs_{Rx}$ from Equation (S1). PV_{Rx} was computed using PV volumes segmented from contrast-enhanced cone-beam CT performed during the planning procedure, a soft tissue density of 1.04 g/cm^3, and Equation (S2). $Liver_{Rx}$ was computed using whole liver volumes segmented from either diagnostic magnetic resonance imaging or CT and Equation (S3).

LSF_{planar} was used for all clinical dosimetry except in a small number of cases where LSF_{SPECT} was used instead (n=11). The limit for $Lungs_{Rx}$ ($Lungs_{max}$) was kept at 30 Gy while the $Liver_{Rx}$ limit ($Liver_{max}$) was set at 120 Gy or less except for a small number of cases where 120 Gy was exceeded. When LSF_{SPECT} was used for the clinical treatment plan, a more conservative $Lungs_{max}$ of 20 Gy was used based on guidance in the literature [3]. Target PV_{Rx} was determined by a combination of factors, including cancer type, prior therapy, $Lungs_{max}$, $Liver_{max}$, among others. For HCC cases, a PV_{Rx} of at least 400 Gy was generally targeted if feasible. Radiation segmentectomy cases often enabled PV_{Rx} to be larger than 400 Gy. Non-HCC cases generally used PV_{Rx} closer to 200 Gy depending on the specific cancer type.

Equations (S1-S3) can be used to derive two versions of a maximum achievable PV dose (PV_{max}) on the basis of allowing $Lungs_{Rx}$ to reach $Lungs_{max}$ or $Liver_{Rx}$ to reach $Liver_{max}$. Equation (S1) is re-arranged to relate the AA value associated with reaching $Lungs_{max}$, while Equation (S3) is re-arranged to relate the AA value associated with $Liver_{max}$. Then the new equations for AA can be combined with Equation (S2) to produce the following:

$$LSF\ and\ LMD_{max}: PV_{max}\ (Gy) = \frac{LMD_{max}(Gy)\times Mass_{lungs}\ (kg)}{PV\ Mass\ (kg)}\cdot\frac{(1-LSF)}{LSF} \tag{S4}$$

$$Liver_{max}: PV_{max}\ (Gy) = \frac{Liver_{max}(Gy) \times Mass_{liver}\ (kg)}{PV\ Mass\ (kg)} \tag{S5}$$

Equations (S4,S5) outlined above are the same as Equations (1,2) in the main manuscript. Building off of Equations (S4,S5), the LSF value that corresponds to PV_{max} in Equation (S4) equaling PV_{max} in Equation (S5) can also be derived. This is the minimum LSF value (LSF_{min}) at which $Lungs_{max}$ starts to limit PV_{max} instead of $Liver_{max}$. After setting Equation (S4) equal to Equation (S5), PV mass is canceled out since it is common to both equations, and then the equations can be re-arranged to solve for LSF (LSF_{min}):

$$LSF_{bound}(\%) = \frac{1}{(TTR \times ratio_{liver_{lungs}}) + 1} \times 100 \tag{S6}$$

Equation (S6) is the same as Equation (3) in the main manuscript. In Equation (S6), the ratio of liver mass ($mass_{liver}$) to lungs mass ($mass_{lungs}$) has been converted to a new variable termed $ratio_{liver\_lungs}$. The ratio of dose thresholds between liver and lungs ($Liver_{max}/Lungs_{max}$) is termed the toxicity threshold ratio (TTR). TTR and $ratio_{liver\_lungs}$ combine to uniquely determine LSF_{bound} for any given patient and case.

As discussed in the main text, modified versions of $Liver_{Rx}$, $Lungs_{Rx}$, and PV_{Rx} were computed in this study relative to those used in clinical treatment plans. This was necessary because if $LSF_{SPECT} < LSF_{planar}$ (nearly all cases), $Liver_{Rx}$ and PV_{Rx} will increase while $Lungs_{Rx}$ will decrease relative to the values computed clinically using LSF_{planar}. Because less of the AA is estimated to go to the lungs with a lower LSF, liver and PV dose increase [2] (see Equation S3). Using a simple example, consider an AA of 3.0 GBq, LSF_{planar} of 10%, LSF_{SPECT} of 3%, liver volume of 2000 cc, PV size of 200 cc, and lung mass of 1-kg. Using LSF_{planar}: $Liver_{Rx}$, PV_{Rx}, and $Lungs_{Rx}$ would be 64.5, 645, and 14.9 Gy, respectively. Using LSF_{SPECT}: the dosimetry values are

69.5, 695, and 4.5 Gy. Since the purpose in this study was to directly analyze LSF_{bound} relative to LSF_{SPECT}, dosimetry using LSF_{SPECT} was necessary to compute clinical TTR and LSF_{bound} values.

Additional Results

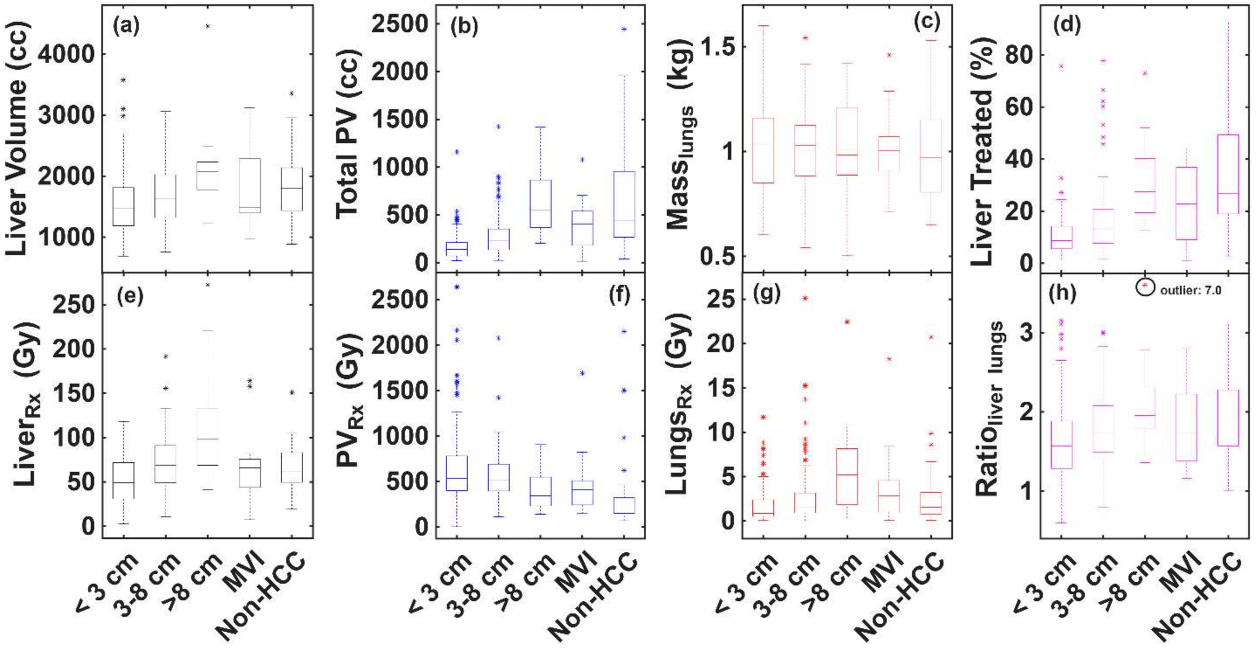

Figure S1. Boxplots showing a) liver volume, b) total PV, c) $mass_{lungs}$, d) % liver treated, e) $Liver_{Rx}$, f) PV_{Rx}, g) $Lungs_{Rx}$, and h) $ratio_{liver\_lungs}$ for the 298 clinically treated cases in this study. The data are separated by cancer type and tumor size (HCC: < 3 cm, 3-8 cm, >8 cm, MVI; non-HCC). The median (line), IQR (box), whiskers (± 3σ), and outliers (> 3σ, *) are all plotted. In (h), a single outlier case with $ratio_{liver\_lungs}$ of 7.0 is plotted at a value of 3.6 to allow for a more appropriate y-axis scale to be used.

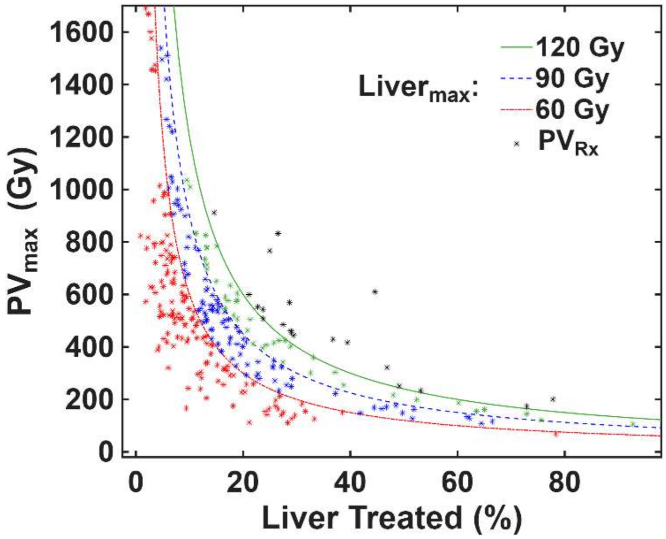

Figure S2. PV_{max} as a function of % liver treated for three different $Liver_{max}$ thresholds of 60, 90, and 120 Gy. In these plots, there is no consideration for $Lungs_{max}$ limiting PV_{max} to a lower value than those shown in the plot computed using $Liver_{max}$. PV_{Rx} values for the 298 clinical cases in this study are also plotted for comparison and separated by color using their categorization for $Liver_{max}$ value from clinical $Liver_{Rx}$. For clarity, a small number of cases with very high PV_{Rx} beyond the y-axis limit are not shown in the figure.

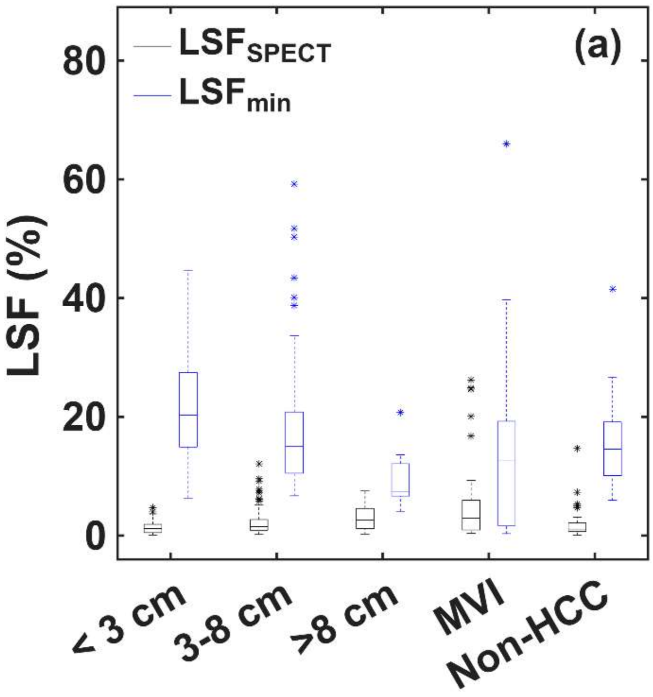

Figure S3. Clinical LSF (left, black) and LSF_{bound} (right, blue) boxplots using LSF_{SPECT} data.

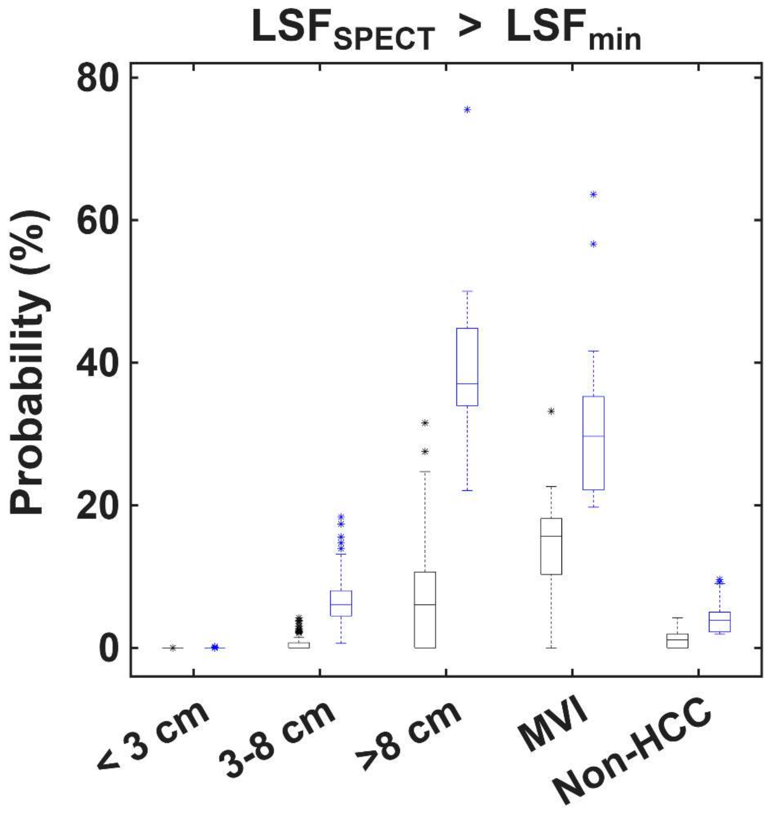

Figure S4. Boxplots showing distributions for $P_{LSF>LSFbound}$ from each of the 5 categories of cases in this study. Data for two versions of LSF_{bound} are included: left (black) is clinical LSF_{bound} computed using the clinical parameters for each case with a $Lungs_{max}$ of 20 Gy for LSF_{SPECT}, right (blue) is LSF_{bound} computed using the maximum TTR for each tumor category (Table 2). The probability estimates were computed from the respective probability density functions for each category of cases that were derived from clinical LSF distributions [2].

Table S1. ROC Analysis: Prospective Use of LSF_{min}

	All Cases	HCC < 3 cm	HCC 3-8 cm	HCC >8 cm	MVI	Non-HCC	Best P_{thresh}[#]
	LSF_{bound}@$Liver_{max}$ = 60 Gy						
$LSF_{SPECT} > LSF_{bound}$ (*n*)	6	0	1	0	4	1	6
P_{thresh} (%)*	0.5	0	0.5	26.6	16.6	2.0	–
Specificity	0.81	1.0	0.91	1.0	0.46	0.98	0.92
Accuracy	0.81	1.0	0.91	1.0	0.54	0.98	0.93
	LSF_{bound}@$Liver_{max}$ = 90 Gy						
$LSF_{SPECT} > LSF_{bound}$ (*n*)	9	0	2	1	5	1	9
P_{thresh} (%)	2.3	0	2.3	43.1	17.2	4.7	–
Specificity	0.87	1.0	0.92	1.0	0.09	0.98	0.90
Accuracy	0.87	1.0	0.92	1.0	0.25	0.98	0.90
	LSF_{bound}@$Liver_{max}$ = 120 Gy						
$LSF_{SPECT} > LSF_{bound}$ (*n*)	13	0	3	3	5	2	13
P_{thresh} (%)	2.3	0.3	2.3	10.9	18.1	3.9	–
Specificity	0.66	1.0	0.62	0.72	0.09	0.78	0.73
Accuracy	0.67	1.0	0.63	0.76	0.25	0.78	0.74

*Max probability threshold where sensitivity still = 1 (negative predictive value = 1)
[#]Results combined using optimal P_{thresh} for each tumor category

Table S1 shows results from the ROC analysis for accurately predicting cases with $LSF_{SPECT} > LSF_{bound}$ at three specific $Liver_{max}$ values of 60, 90, and 120 Gy. At a low $Liver_{max}$ of 60 Gy, 6 cases had $LSF_{SPECT} > LSF_{bound}$. Under these conditions with a relatively low $Liver_{max}$, a

small P_{thresh} was required to ensure no false negative results in all groups except for HCC >8 cm and tumors with MVI. A global threshold of 0.5% across all cases still produced a specificity and accuracy of 81%, indicating a clinically acceptable number of false positives. Using a more specific P_{thresh} for each tumor group enabled improved predictive performance overall. Nearly all P_{thresh} values produced equivalent results for HCC < 3 cm tumors at the three $Liver_{max}$ values tested. This means that so long as it is not expected to use an unexpectedly high $Liver_{max}$ for such cases, $P_{LSF>LSFbound} > 0$ represents relevant risk and a patient-specific, MAA-based LSF would be required. Non-HCC cases were also predicted with clinically useful accuracy, but at slightly higher P_{thresh} relative to HCC < 3 cm and HCC 3-8 cm tumor groups. HCC >8 cm and MVI tumor groups both generally required high P_{thresh} values to avoid high numbers of false positives. From a clinical use standpoint, such high P_{thresh} values would likely be difficult to employ while maintaining confidence in reliable pre-treatment predictions.

Table 2 in the main text includes parameters for five example cases from each tumor category. These example cases can provide perspective on how the pre-treatment approach to using LSF_{bound} for treatment planning could be employed. Case 1 is a small HCC tumor located in segment 7, leading to a PV size of 95 cm^3. A liver volume of 1120 cm^3 and lung mass of 1.08 kg produced a small $ratio_{liver\_lungs}$ of 1.08. The PV_{Rx} was 505 Gy for radiation segmentectomy and led to a $Liver_{Rx}$ of 42 Gy. This produced a TTR of 2.1 and when combined with a $ratio_{liver\_lungs}$ of 1.08 yielded an LSF_{bound} of 30.5%. Since this was a small HCC tumor (< 3 cm), $P_{LSF>LSFbound}$ was 0%. There would be absolutely no risk for Case 1 to have a clinical LSF > LSF_{bound}, so no MAA injection was needed for this case. For a case such as this, PV_{Rx} could be increased dramatically up to >1500 Gy ($Liver_{Rx}$ of >120 Gy) and still keep LSF_{bound} well above 5% and therefore $P_{LSF>LSFbound}$ still = 0%. The actual LSF_{SPECT} for this case was 2.7%.

Cases 2 involved a larger liver and a larger 6.4 cm HCC tumor in segment 4. Case 2 had a larger PV volume of 330 cm^3 and a PV_{Rx} of 410 Gy. The $Liver_{Rx}$ was still low at 57 Gy due the larger liver size relative to Case 1. All these parameters produced a $ratio_{liver\_lungs}$ of 2.06 and LSF_{bound} of 14.6%. For this tumor category (HCC 3-8 cm), $P_{LSF>LSFbound}$ was also 0%. No patient-specific, MAA-based LSF would be needed for this case either. The actual LSF_{SPECT} was 1.2%. PV_{Rx} (and $Liver_{Rx}$) could have been increased in this case as well and still kept $P_{LSF>LSFbound}$ at or near 0%.

Case 3 involved a large 9.6 cm HCC tumor in segments 7 and 8. The PV volume was also much larger at 780 cm^3 and the PV_{Rx} was below 400 Gy at 305 Gy. This produced a $Liver_{Rx}$ of 113 Gy. The $ratio_{liver\_lungs}$ was 2.46 and LSF_{bound} was 6.7%. This is a relatively low LSF_{bound}, created by the combination of a larger liver volume, lung mass < 1 kg, and $Liver_{Rx}$ approaching 120 Gy. Since this tumor category (HCC >8 cm) maintains an increased risk for high LSF, $P_{LSF>LSFbound}$ was nonzero at 10%. Each institution could decide how to approach these results for Case 3. But our recommendation would be that a 10% chance for clinical LSF to exceed LSF_{bound} means a patient-specific MAA-based LSF is warranted and may provide clinical benefit. Only after assessing the MAA-based LSF would the most informed treatment planning approach for this case be possible. The clinical LSF_{SPECT} for this case was 7.5% and indeed exceeded LSF_{bound}.

Case 4 was another large HCC tumor in segment 8 but with MVI present. This case also included a much smaller than expected liver volume considering the size of the HCC tumor (9 cm). A less aggressive PV_{Rx} of 150 Gy produced a $Liver_{Rx}$ of only 62 Gy despite the large PV size of 540 cm^3 and small liver volume of 1280 cm^3. The small $ratio_{liver\_lungs}$ of 1.32 produced an LSF_{bound} of 19.6%. For cases without MVI this would yield $P_{LSF>LSFbound}$ near 0% but with MVI the probability was 13%. The likely recommendation would therefore be to proceed with obtaining

a patient-specific LSF for this case as well. The clinical LSF_{SPECT} was 9.3%, well below LSF_{bound}. However, the clinical PV_{Rx} for this case was low at only 150 Gy. Had this case been analyzed pre-treatment with the intention for a higher PV_{Rx} of 300 Gy, the $Liver_{Rx}$ would have increased to 124 Gy and LSF_{bound} would have decreased to 10.9%. Generally, most all HCC >8 cm tumors and tumors with MVI should require patient-specific LSF considering their increased risk for high LSF and lower LSF_{bound}.

The final case was a 4.0 cm non-HCC (colorectal cancer metastasis) in segment 4. The PV volume was 360 cm^3 and the PV_{Rx} was 310 Gy. This produced a small $Liver_{Rx}$ of 53 Gy due to the relatively large liver volume of 2100 cm^3. The $ratio_{liver\_lungs}$ was 2.65 and LSF_{bound} was 12.6%. For the non-HCC tumor category this produced a $P_{LSF>LSFbound}$ of 3%. This probability is nonzero but still quite low and less than 5%. The clinical utility of a patient-specific, MAA-based LSF for this case is likely very small so long as single-compartment dosimetry can be used for treatment planning. It would likely be safe to proceed in this case without an MAA injection unless a more aggressive PV_{Rx} would have been considered pre-treatment.

Additional Discussion

Until more accurate lung dosimetry from post-therapy imaging such as positron emission tomography (PET)/CT [4] or Monte-Carlo corrected SPECT/CT [5] can be sufficiently validated, methods for documenting absorbed lung dose in ^{90}Y-SIRT remains limited. A related topic is an appropriate $Lungs_{max}$ when using LSF_{SPECT} in place of LSF_{planar}. Unfortunately, there is currently no "ground truth" for LSF or $Lungs_{Rx}$ in ^{90}Y-SIRT. There have been examples in the literature of cases with estimated $Lungs_{Rx}$ < 30 Gy that still led to radiation pneumonitis [6]. All analyses in this study considered a lower $Lungs_{max}$ of 20 Gy with LSF_{SPECT} relative to the traditional 30 Gy with LSF_{planar}. This was based on published guidance in the literature [3]. Notably, to reach 20 Gy

$Lungs_{Rx}$ when using LSF_{SPECT}, a very high AA would be needed for most cases because LSF_{SPECT} is so often very low. The AA values required to increase PV_{Rx} up to PV_{max} for the cases in this study would have produced $Lungs_{Rx}$ >15 Gy in only 7 (2%) cases, with only 4 (1%) reaching the 20 Gy $Lungs_{max}$ limit. These assessments align with the other results in this study: liver dose thresholds dictate PV_{max} much more often so that $Lungs_{Rx}$ when using LSF_{SPECT} tends to remain well below 20 Gy.

References

1. Gabr A, Ranganathan S, Mouli SK, Riaz A, Gates VL, et al. Streamlining radioembolization in UNOS T1/T2 hepatocellular carcinoma by eliminating lung shunt estimation. J Hepatol. 2021; 72:1151-1158.
2. Thomas MA, Alamuri T, Lee RC, Giardina D, Karageorgiou J, et al. Modeling Clinical Relevance and Risk in Yttrium-90 Radioembolization of the Liver: Lung Shunt Fraction Variability According to Imaging Modality, Cancer Type, and Tumor Size. J. Vasc. Interv. Radiol. 2025; DOI: 10.1016/j.jvir.2025.09.006
3. Kappadath SC, Lopez BP, Salem R, Lam MGEH. Reassessment of the lung dose limits for radioembolization. Nucl Med Commun. 2021; 42:1064–75.
4. Stella M, van Rooij R, Lam MGEH, de Jong HWAM, Braat AJAT. Lung dose measured on post-radioembolization ^{90}Y-PET/CT and incidence of radiation pneumonitis. Journal of Nuclear Medicine. 2022; 63:1075–80.
5. Kunnen B, van der Velden S, Bastiaannet R, Lam MGEH, Viergever MA, de Jong HWAM. Radioembolization lung shunt estimation based on a ^{90}Y pretreatment procedure: A phantom study. Med Phys. 2018; 45:4744–53.

6. Kis B and Gyano M. Radiation Pneumonitis After Yttrium-90 Radioembolization: A Systematic Review. J. Vasc Interv. Radiol. 2025; 36: 207-218.
